# Ma-Huang-Fu-Zi-Xi-Xin Decoction for Allergic Rhinitis: A Systematic Review

**DOI:** 10.1155/2018/8132798

**Published:** 2018-02-05

**Authors:** Juan Zhong, Dan Lai, Yun Zheng, Gang Li

**Affiliations:** ^1^Hearing Center/Hearing & Speech Science Laboratory, Department of Otorhinolaryngology-Head and Neck Surgery, West China Hospital, Sichuan University, Chengdu 610041, China; ^2^Department of Otolaryngology-Head and Neck Surgery, The Affiliated Hospital of Southwest Medical University, Luzhou 646000, China

## Abstract

**Background:**

The treatment effects and safety of Ma-Huang-Fu-Zi-Xi-Xin decoction for patients with allergic rhinitis have yet to be clarified.

**Objectives:**

The aim of this study is to evaluate the effects and safety of Ma-Huang-Fu-Zi-Xi-Xin decoction in patients with allergic rhinitis.

**Methods:**

We searched PubMed, EMBASE (Excerpta Medical Database), Cochrane Library, Chinese Cochrane Centre's Controlled Trials Register Platform, Wanfang Chinese Digital Periodical and Conference Database, China National Knowledge Infrastructure (CNKI) Database, and VIP Chinese Science and Technique Journals Database to collect randomized controlled trials of Ma-Huang-Fu-Zi-Xi-Xin decoction (MHFZXXD) for allergic rhinitis (AR) prior to May 8, 2017. RevMan 5.3 software was used to conduct a meta-analysis. GRADE methodology was applied to evaluate the evidence quality for each outcome.

**Results:**

Six randomized controlled trials (RCTs) involving 576 participants (10–78 years old) were included. A meta-analysis revealed that the overall effect of MHFZXXD for AR was not better than western medical treatment (RR: 0.41; 95% CI: 0.26–0.65; *P* = 0.0001) for all included studies. However, the evidence quality of these western medical intervention studies was low or very low due to a high risk of bias, small sample sizes, and poor-quality design.

## 1. Introduction

Allergic rhinitis (AR) is defined as chronic inflammation of the nasal mucous membrane, typically induced by immunoglobulin E- (IgE-) mediated sensitization to environmental allergens. These include dust, domestic animals, pollens, and molds. AR is defined by the onset of two or more of the following symptoms: nasal discharge, sneezing, nasal itching, and congestion, all of which interfere with activities of daily living, disrupt regular sleep patterns, or exert a negative influence on the patient's social life or intellectual performance.

The incidence of AR is approximately 20%–40% in the US population [1]. A survey of the worldwide pediatric population showed that the morbidity of AR was 10%–20% in 2015 [2]. Subcutaneous injection immunotherapy and sublingual immunotherapy [3] are common treatment modalities for this disease, but the effectiveness of those therapies is in doubt and remains to be demonstrated conclusively.

As a classical herbal medicine formula for AR, Ma-Huang-Fu-Zi-Xi-Xin decoction (MHFZXXD) and other herbal medicine formulas as effective interventions have been recommended in a Clinical Guideline for alleviation of symptoms of AR in 2015 [4]. According to Traditional Chinese Medicine (TCM) theory, the pathogenesis of AR falls into one of two categories: excess or deficiency syndromes. In general, excess syndrome stands for a series of symptoms that are always caused by an invasion, an exogenous pathogenic factor, flaring of visceral fire, stagnancy of the blood, or accumulation of phlegm/dampness. Deficiency syndrome is generally associated with insufficiency of viscera. MHFZXXD is thought to act by strengthening bodily resistance with the effect of relieving the “superficies syndrome.” Stated another way, the therapy is designed to adjust a skewed state and to maintain a balance between yin and yang.

MHFZXXD is described in a TCM classical document called the Treatise on Febrile Disease, written at the beginning of the third century. According to the Treatise, MHFZXXD contains three herbal medicines, Mahuang (Herba Ephedrae), Fuzi (Radix Aconiti Praeparata), and Xixin (Herba Asari). Many believe in the long-term clinical effects of MHFZXXD for AR [5]. An animal experimental study suggested that MHFZXXD is more effective in treating AR, with better efficacy than the Xiaoqinglong decoction for reducing histamine content in blood and recovery of nasal mucosa in guinea pigs [6].

At least three reviews of herbal medicine therapy for AR have been located [7–9]. However, these reviews did not consider MHFZXXD. With the publication of a fair number of trials on MHFZXXD for AR in recent years, there is an urgent need for a systematic review to summarize the evidence from all available studies of MHFZXXD. Thus, the aim of this review was to evaluate critically the current state of evidence from RCTs on the use of MHFZXXD in patients of AR, according to the guidelines set down in the Cochrane Handbook.

## 2. Methods

### 2.1. Search Strategy

A systematic search for related published and ongoing trials was performed on May 8, 2017. The languages of the studies were limited to Chinese or English.

### 2.2. Electronic Searches

A total of six databases were searched: PubMed (1992 to May 8, 2017), EMBASE (Excerpta Medical Database) (1992 to May 8, 2017), Cochrane Library (Issue 9 of May 8, 2017), Chinese Cochrane Centre's Controlled Trials Register Platform (up to May 8, 2017), Wanfang Chinese Digital Periodical and Conference Database (1997 to May 8, 2017), China National Knowledge Infrastructure (CNKI) Database (1992 to May 8, 2017), and VIP Chinese Science and Technique Journals Database (1992 to May 8, 2017). Besides, Chinese Clinical Trial Registry Center was also retrieved for ongoing trials.

Search terms were as follows:

Ma Huang Fu Zi Xi Xin decoction
Ma-Huang-Fu-Zi-Xi-Xin decoction
MHFZXXD
Chinese Herbal Medicine
Integrated Chinese and Western medicines
Integrated traditional and Western medicine
(1)–(6)/OR
Allergic rhinitis
Rhinallergosis
AR
(8)–(10)/OR
(7) AND (11)

### 2.3. Other Search Resources

References of related identified publications were checked for additional trials, and we contacted authors by e-mail or telephone for additional data where necessary.

### 2.4. Study Types

Prospective randomized controlled trials (RCTs) of Ma-Huang-Fu-Zi-Xi-Xin decoction versus placebo or conventional medicine were included in this review. Observational studies, case reports, case series, qualitative studies, uncontrolled studies, and studies with no randomization-control design were excluded.

### 2.5. Participants

Patients presenting with seasonal AR or perennial AR were all included. Allergic rhinitis merged with allergic asthma or allergic conjunctivitis and other allergic diseases were excluded. This was done because targeted drug combination methods in these studies could not be used to compare effects.

### 2.6. Interventions

We compared MHFZXXD with conventional medicine or placebo regimens. MHFZXXD compared with other types of Chinese herbal medicine formulas such as Gui-Zhi decoction, Bu-Zhong-Yi-Qi decoction, and Yu-Ping-Feng decoction was excluded.

Interventions considered for experimental groups versus control groups were as follows:

(1) MHFZXXD versus conventional medicine, including chlorpheniramine, 1% ephedrine nose drops, cortisone, budesonide nasal spray, ebastine tablets, loratadine tablets, or other commonly used efficacy drugs.

(2) MHFZXXD combined with conventional medicine versus conventional medicine.

(3) MHFZXXD combined with other complementary therapies versus other complementary therapies.

(4) MHFZXXD versus placebo.

We excluded studies or trials with MHFZXXD performed as a part of complex interventions versus other types of regimens, for example, Ma-Huang-Fu-Zi-Xi-Xin decoction plus another herbal medicine formula versus acupuncture therapies.

### 2.7. Outcome Measures

Trials were required to include as outcome measures either relief of symptoms of AR or evaluation of the efficacy of MHFZXXD in AR. Other important clinical outcomes included recurrence rate, influence on quality of life, improvement in symptom scoring, and adverse events.

The efficacy of MHFZXXD for AR, improvement in quality of life, and improvement of symptom scoring were set as primary outcomes. Recurrence rate and adverse events (including dry mouth, headache, hypersomnia, palpitations, and gastrointestinal discomfort) were set as secondary outcomes.

### 2.8. Study Selection, Data Extraction, and Quality Assessment

#### 2.8.1. Study Selection

Two authors (Juan Zhong and Dan Lai) scanned titles and abstracts of all papers. A determination was then made as to whether the studies met our inclusion criteria. Conflicts were resolved by discussion.

#### 2.8.2. Data Extraction and Management

Raw data of included papers containing the details of authors, the publication information, and design information of the original study were separately extracted by three authors (Juan Zhong, Gang Li, and Dan Lai).

### 2.9. Assessment of Risk of Bias

The risk of bias was assessed according to the Cochrane Handbook for Systematic Reviews of Interventions. The latest version of this tool was updated in March 2011, version 5.1.0 (http://www.handbook.cochrane.org/) [10]. Two authors assessed the risk of bias according to this tool. All disagreements were resolved by the way of discussion. Risk of bias items included the following: randomization sequence generation, allocation concealment, blinding of participants or healthcare providers, detection bias, incompleteness bias, reporting bias, and other biases.

### 2.10. Data Analysis

Review Manager (RevMan) software version 5.3 was applied to pool our data to perform the meta-analysis. GRADE profile software was used to record the results of the GRADE evidence rating.

Risk ratio (RR) was chosen for dichotomous data (efficacy, recurrence rate, and adverse events). Confidence interval (CI) was set at 95%, and *P* < 0.05 was defined as statistically significant. *I* ^2^ values were used to assess interstudy heterogeneity. When *I* ^2^ > 75%, considerable heterogeneity was conformed, whereupon a random-effects model was applied [10]. We pooled trials when the intervention forms of those studies were adequately similar. Specific subgroups were analyzed according to similar intervention forms or similar design.

## 3. Results

### 3.1. Study Description

#### 3.1.1. Search Results

We initially identified a total of 53 trials using the specific search strategy in our protocol. No unpublished or ongoing studies were found. After reviewing titles, abstracts, and keywords, twenty-six papers were excluded for failure to conform to inclusion criteria. Eighteen duplicated texts were excluded, as well as nine studies that had initially appeared to meet our inclusion criteria. After the full text was read, six studies finally met our inclusion criteria. The study selection process is outlined in Figure S1.

### 3.2. Included Studies and Excluded Studies

There were six Chinese language studies comprising 576 participants aged 10–78 years [11–17], published between 2006 and 2015. Interventions in these studies were MHFZXXD versus conventional therapy (western medicine therapy): three [13, 14, 16] compared MHFZXXD to loratadine tablets, and the remainder [11, 12, 15] compared MHFZXXD to mixed western medicine therapy. There were six RCTs [11–16]. Effective rate indicator was regarded as the most important outcome measure in all trials. Two trials [13, 14] recorded adverse events, and one study [16] reported six-month recurrence rate. No participant withdrawal information was reported in these studies. No indicator of the influence on life quality as an outcome measure was reported in those studies. Characteristics of the six included studies are displayed in Table S1.

In Zhu's work (2009) [17], participants were allocated to experimental and control groups by visit sequence. Thus, this study could be defined as a semirandomized trial. Hu [18] divided patients according to predetermined therapy rather than random assignment. Therefore, this trial could not be considered as an RCT. In the third article published in 2015 [19], another Chinese herbal medicine preparation named angelia rhinitis, drop pills were determined as the control group intervention. This kind of comparison does not conform to the inclusion criteria. Therefore, these three studies were excluded. Characteristics of excluded three studies are displayed in Table S2.

### 3.3. Risk of Bias

#### 3.3.1. Allocation (Selection Bias)

Six studies were designed as randomized controlled trials [11–16], of which three studies [13, 15, 16] randomly divided all participants using a random number table tool. However, the authors of these studies failed to report details of the patient allocation technique. Hence, a high risk of bias could be defined in these trials. The randomization method and allocation concealment details of patient distribution of three other studies [11, 12, 14] were not mentioned in the original text. Therefore, there may be a high risk of selection bias in these trials.

#### 3.3.2. Blinding (Performance Bias and Detection Bias)

No blinding method was mentioned in any of the six included studies. There might present a high risk of performance and detection bias.

#### 3.3.3. Incomplete Outcome Data (Attrition Bias)

There might be unclear attrition bias in the six studies due to absence of sample size calculation. No cases lost case or withdrawal information was provided in these studies.

#### 3.3.4. Selective Reporting (Reporting Bias)

Unclear reporting bias was detected in the six trials due to absence of protocol. None of the trials declared a clinical trial registration number.

#### 3.3.5. Other Potential Sources of Bias

No other potential sources of bias were found.

The risk of bias graph of authors' judgements concerning included studies is displayed in Figure S2.

The risk of bias summary of authors' judgements concerning included studies is displayed in Figure S3.

### 3.4. Effects of Interventions

Six studies [11–16] comprising 576 participants were included in this review. Intervention forms used in RCTs can be classified into four types. Therefore, we performed intervention-specific subanalyses of RCTs in line with types of intervention form. The efficacy of three trials with MHFZXXD versus loratadine tablets was also analyzed. Adverse events (safety) and recurrence of MHFZXXD for AR were also analyzed.

The efficacy of six RCTs comparing particular interventions and MHFZXXD is presented in Figure S4. Adverse events (safety) evaluation of MHFZXXD is presented in Figure S5. The recurrence risk is presented in Figure S6.

### 3.5. MHFZXXD versus Loratadine Tablets (Three RCTs)

Three RCTs [13, 14, 16] tested the efficacy of MHFZXXD compared with loratadine tablets. We pooled these trials using RevMan 5.3. MHFZXXD was not superior to loratadine tablets (RR: 0.48; 95% CI: 0.25–0.91; *P* = 0.02; Figure S4).

### 3.6. MHFZXXD versus Chlorpheniramine Combined with 1% Ephedrine Nose Drops and Cortisone

One clinical trial published in 2006 [11] assessed the effectiveness of MHFZXXD versus these medications. There was no statistically significant difference (RR: 0.77; 95% CI: 0.25–2.4; *P* = 0.65; Figure S4).

### 3.7. MHFZXXD versus Budesonide Nasal Spray and Ebastine Tablets

One trial [12] published in 2011 made this comparison. The control group intervention had a better effect than the MHFZXXD group (RR: 0.31, 95% CI: 0.11–0.86; *P* = 0.03; Figure S4).

### 3.8. MHFZXXD versus Dexamethasone, Gentamicin, and Chymotrypsin

Yao and Li's work [15] published in 2015 performed this comparison. RevMan software analysis revealed that the control group performed better than the MHFZXXD group (RR: 0.18; 95% CI: 0.04–0.77; *P* = 0.02; Figure S4).

### 3.9. Adverse Events

Two studies [13, 14] reported the details of adverse events. Sha [13] noted that two participants (4.00%) in the experimental group presented with transient dryness of the mouth. The symptom abated when the patient stopped MHFZXXD. Nine patients (18.0%) reported adverse events in the control group: five with headache, two with hypersomnia, and two with gastrointestinal complaints. These symptoms all abated following treatment. The paper wrote by Wang [14] reported that no adverse events in the experimental group had happened, while three patients (one had palpitation and two had mouth dryness) and thus 5.7% of participants have had drug side effect in control group. We pooled these data: MHFZXXD had better safety than loratadine tablets (RR: 0.20; 95% CI: 0.05–0.75; *P* = 0.02; Figure S5).

### 3.10. Recurrence Rate

One study published in 2015 (Ye and Lin [16]) reported six-month follow-up. There was no statistically significant difference (RR: 0.38; 95% CI: 0.11–1.29; *P* = 0.12; Figure S7). This is the only trial that reported follow-up conditions, including patients whose four AR symptoms recurred at three and six months, respectively. Two patients in experimental group and five patients in control group recurred at the third month. 3 versus 8 participants recurred at 6 months.

### 3.11. Quality of Evidence

The quality of evidence for outcome measures according to the GRADE system is displayed in Tables S2, S3, and S4.

## 4. Discussion

### 4.1. Overview of Findings

To the best of our knowledge, this is the first meta-analysis of MHFZXXD therapy for AR patients. Six trials were included in our review. All control group interventions can be characterized as western medical therapy. MHFZXXD was not inferior to western medicine in improving patients' AR symptoms. However, the data extracted from six trials had small simple size and poor quality measures, according to the GRADE methodology (Tables S2, S3, and S4).

None of our included studies used validated questionnaire and scales, including rhinoconjunctivitis, Quality Of Life Questionnaire (RQLQ), and visual analogue scale (VAS), to measure melioration of AR severity or disability, despite the fact that these scales are recommended by the 2015 Clinical Guideline [4]. RQLQ is a tool used to evaluate the quality of life of AR patients. VAS is used to assess the severity of symptoms of AR. Daily life quality evaluation and severity assessment for participants were not performed in the included trials.

Despite our use of validated documents supporting diagnostic criteria and effectiveness assessment criteria in this review, nonuniform diagnostic approach or standard of efficacy evaluation might influence outcomes and results. It might be difficult to employ the same diagnosis and effectiveness assessment criteria for each trial, as these criteria vary with each update. Nevertheless, we employed the latest or most recently published criteria.

Prior to our study, there have been at least three systematic reviews [7–9] focusing on the efficacy of Chinese herbal medicine or herbal medicine as a treatment for AR. However, none of these reviews included MHFZXXD as an experimental intervention. Since MHFZXXD has been widely used for treating AR in China in many years, we believed there was an urgent need to update the systematic review to present an objective evaluation of this intervention.

### 4.2. Quality of Evidence

The six included studies were prospective, randomized, placebo-controlled studies. However, just three studies [13, 15, 16] mentioned the method of randomization. No study stated whether the design was double-blinded. Therefore, there is a potential risk of measurement and implementation bias. No trial mentioned allocation concealment or any concealment method.

It was not clear whether incomplete outcome data were adequately addressed, as no trial reported drop-out rates. The risk of incomplete outcome data bias in these six studies is therefore unclear. The risk of selective reporting bias is unclear as we do not know whether these six trials reported all prespecified, expected results. Therefore, the quality of evidence for the outcome measurement was low for one outcome and very low for six outcomes, according to the GRADE system.

### 4.3. Potential Biases in the Review Process

No ongoing trials were found. The conclusion of this review was drawn from the six trials, which comprised a limited number of participants. More studies and high-quality trials should be included in future reviews.

In addition, dexamethasone plus gentamicin and chymotrypsin are not used for treatment of allergic rhinitis alone. The diagnosis of allergic rhinitis in Yao and Li's study was questionable, even though we have already contacted the authors of that paper to confirm that this clinical experiment was real and reliable. Another crucial issue is that investigation duration varies in those studies. The treatment course in those studies varies from 3 weeks to 3 months. These critical differences might be an important factor with direct influence on the efficacy assessment and might be a factor leading to bias.

## 5. Conclusion

In conclusion, current evidence suggests that MHFZXXD alone cannot be recommended for the treatment of AR. This conclusion might rest on a negative summary proof of MHFZXXD or on publication bias or study-design limitations of the included studies. There are insufficient data to state that MHFZXXD is safe and reliable due to the small number of trials reporting adverse events. Therefore, larger sample size and rigorously designed studies are necessary to determine conclusively a definitive association between MHFZXXD and AR.

### 5.1. Implications for Practice

There is no support for the use of MHFZXXD for the treatment of AR.

### 5.2. Implications for Research

There is an urgent need for double-blind, prospective, randomized, placebo-control trials of MHFZXXD as a treatment for AR. Such studies should employ uniform and validated diagnostic criteria, efficacy evaluation criteria, recommended questionnaire, and measurement scales. Long-term follow-up effectiveness and safety studies for MHFFXXD in AR are necessary as well.

## Supplementary Materials

Supplementary Materials Figure S1: flow diagram of study selection process in this systematic review. Figure S2: risk of bias graph of authors' judgements about included studies. Figure S3: risk of bias summary of authors' judgements about included studies. Figure S4: efficacy of 6 RCTs of MHFZXXD versus western medical therapy. Figure S5: safety of MHFZXXD versus western medical therapy. Figure S6: six months of recurrence of MHFZXXD versus western medical therapy. Table S2: quality of evidence for outcome measure of efficacy. Table S3: quality of evidence for outcome measures of safety. Table S4: quality of evidence for outcome measure of recurrence rate. Table S1: characteristics of included studies. Table S2: characteristics of excluded studies.
