# Supplementary material for: Ma-Huang-Fu-Zi-Xi-Xin Decoction for Allergic Rhinitis: A Systematic Review
Source: Evid Based Complement Alternat Med. 2018 Feb 5;2018:8132798. doi: 10.1155/2018/8132798 (PMC5832110; doi:10.1155/2018/8132798)
Supplement: Supplementary Materials — Figure S1: flow diagram of study selection process in this systematic review. Figure S2: risk of bias graph of authors' judgements about included studies. Figure S3: risk of bias summary of authors' judgements about included studies. Figure S4: efficacy of 6 RCTs of MHFZXXD versus western medical therapy. Figure S5: safety of MHFZXXD versus western medical therapy. Figure S6: six months of recurrence of MHFZXXD versus western medical therapy. Table S2: quality of evidence for outcome measure of efficacy. Table S3: quality of evidence for outcome measures of safety. Table S4: quality of evidence for outcome measure of recurrence rate. Table S1: characteristics of included studies. Table S2: characteristics of excluded studies. [file 8132798.f1.zip › 8132798.f1/Table S1. Characteristics of included studies..docx]

| Trials | Sample size(E/C) | Gender(E/C) and age(yr) | Duration | Criteria of diagnose | Criteria of efficacy assessment | Interventions | | Period | Outcome measure | Balance report of baseline |
| --- | --- | --- | --- | --- | --- | --- | --- | --- | --- | --- |
|  |  |  |  |  |  | Experimental group | Control group |  |  |  |
| Lin2006 | 146(76/70) | (50M:26F)/ (47M:23F) 13-68 | Mean5.1 years | 1994 criteria | 1994 criteria | Modified Mahuangfuzixixin Decoction | Chlorpheniramine;1% ephedrine nose drops;cortisone | 3 months | effective rate | No significant differences |
| Lu2011 | 80(40/40) | (18M:22F)/ (19M:21F) 10-45 | 3months-7years | 1997criteria | 1997criteria | Modified Mahuangfuzixixin Decoction | Budesonide nasal spray;Ebastine Tablets | 3 weeks | effective rate | P＞0.05 |
| Sha2012 | 100(50/50) | 64M/36F 18-62 | 0.3-31year | 1997criteria | 1997criteria | Modified Mahuangfuzixixin Decoction | Loratadine Tablets | 28 days | effective rate;adverse event | P＞0.05 |
| Wang2015 | 106(53/53) | (15M:38F)/ (15M:38F) 12-78 | 1-16years | 1997criteria | 1994criteria | Modified Mahuangfuzixixin Decoction | Loratadine Tablets | 21days | effective rate;adverse event | No significant differences |
| Yao2015 | 80(40/40) | (28M:12F)/ (29M:11F) 16-55 | 1-6years | 2009criteria | 2009criteria | Modified Mahuangfuzixixin Decoction | pulverization with 3 drugs (Dexamethasone,gentamicin,and chymotrypsin) | 3 weeks | effective rate;symptom scoring | P＞0.05 |
| Ye2015 | 64(32/32) | (18M:14F)/ (20M:12F) 18-65 | 9-56months | 2009criteria | 2009criteria | Modified Mahuangfuzixixin Decoction | Loratadine Tablets | 28days | effective rate;6 months recurrence rate | P＞0.05 |
