# Supplementary material for: Ma-Huang-Fu-Zi-Xi-Xin Decoction for Allergic Rhinitis: A Systematic Review
Source: Evid Based Complement Alternat Med. 2018 Feb 5;2018:8132798. doi: 10.1155/2018/8132798 (PMC5832110; doi:10.1155/2018/8132798)
Supplement: Supplementary Materials — Figure S1: flow diagram of study selection process in this systematic review. Figure S2: risk of bias graph of authors' judgements about included studies. Figure S3: risk of bias summary of authors' judgements about included studies. Figure S4: efficacy of 6 RCTs of MHFZXXD versus western medical therapy. Figure S5: safety of MHFZXXD versus western medical therapy. Figure S6: six months of recurrence of MHFZXXD versus western medical therapy. Table S2: quality of evidence for outcome measure of efficacy. Table S3: quality of evidence for outcome measures of safety. Table S4: quality of evidence for outcome measure of recurrence rate. Table S1: characteristics of included studies. Table S2: characteristics of excluded studies. [file 8132798.f1.zip › 8132798.f1/Table S2 Quality of evidence for outcome measure of efficacy.docx]

| **Quality assessment** | | | | | | | **Summary of findings** | | | | | **Importance** |
| --- | --- | --- | --- | --- | --- | --- | --- | --- | --- | --- | --- | --- |
|  |  |  |  |  |  |  | **No of patients** | | **Effect** | | **Quality** |  |
| **No of studies** | **Design** | **Limitations** | **Inconsistency** | **Indirectness** | **Imprecision** | **Other considerations** | **Mahuangfuzixixin Decoction** | **Western medicine therapy** | **Relative (95% CI)** | **Absolute** |  |  |
| **RCTs** | | | | | | | | | | | | |
| 6 | randomised trials | serious^1,2^ | no serious inconsistency | no serious indirectness | no serious imprecision | reporting bias^3^ | 23/291 (7.9%) | 55/285 (19.3%) | RR 0.41 (0.26 to 0.65) | 114 fewer per 1000 (from 68 fewer to 143 fewer) | ⊕⊕OO LOW | CRITICAL |
|  |  |  |  |  |  |  |  | 23.5% |  | 139 fewer per 1000 (from 82 fewer to 174 fewer) |  |  |
| **RCTs - MHFZXXD vs. Loratadine Tablets** | | | | | | | | | | | | |
| 3 | randomised trials | serious^1,2^ | no serious inconsistency | no serious indirectness | serious^4^ | reporting bias^3^ | 12/135 (8.9%) | 25/135 (18.5%) | RR 0.48 (0.25 to 0.91) | 96 fewer per 1000 (from 17 fewer to 139 fewer) | ⊕OOO VERY LOW | IMPORTANT |
|  |  |  |  |  |  |  |  | 22% |  | 114 fewer per 1000 (from 20 fewer to 165 fewer) |  |  |
| **RCTs - MHFZXXD vs. Chlorpheniramine combined with 1% ephedrine nose drops and cortisone** | | | | | | | | | | | | |
| 1 | randomised trials | very serious^1,2^ | no serious inconsistency | no serious indirectness | serious^4^ | reporting bias^3,5^ | 5/76 (6.6%) | 6/70 (8.6%) | RR 0.77 (0.25 to 2.4) | 20 fewer per 1000 (from 64 fewer to 120 more) | ⊕OOO VERY LOW | NOT IMPORTANT |
|  |  |  |  |  |  |  |  | 8.6% |  | 20 fewer per 1000 (from 65 fewer to 120 more) |  |  |
| **RCTs - MHFZXXD vs. Budesonide nasal spray and Ebastine Tablets** | | | | | | | | | | | | |
| 1 | randomised trials | very serious^1,2^ | no serious inconsistency | no serious indirectness | serious^4^ | reporting bias^3,5^ | 4/40 (10%) | 13/40 (32.5%) | RR 0.31 (0.11 to 0.86) | 224 fewer per 1000 (from 45 fewer to 289 fewer) | ⊕OOO VERY LOW | NOT IMPORTANT |
|  |  |  |  |  |  |  |  | 32.5% |  | 224 fewer per 1000 (from 45 fewer to 289 fewer) |  |  |
| **RCTs - MHFZXXD vs. Dexamethasone, gentamicin, and chymotrypsin** | | | | | | | | | | | | |
| 1 | randomised trials | very serious^1,2^ | no serious inconsistency | no serious indirectness | serious^4^ | reporting bias^3,5^ | 2/40 (5%) | 11/40 (27.5%) | RR 0.18 (0.04 to 0.77) | 226 fewer per 1000 (from 63 fewer to 264 fewer) | ⊕OOO VERY LOW | NOT IMPORTANT |
|  |  |  |  |  |  |  |  | 27.5% |  | 226 fewer per 1000 (from 63 fewer to 264 fewer) |  |  |
