# Supplementary material for: Ma-Huang-Fu-Zi-Xi-Xin Decoction for Allergic Rhinitis: A Systematic Review
Source: Evid Based Complement Alternat Med. 2018 Feb 5;2018:8132798. doi: 10.1155/2018/8132798 (PMC5832110; doi:10.1155/2018/8132798)
Supplement: Supplementary Materials — Figure S1: flow diagram of study selection process in this systematic review. Figure S2: risk of bias graph of authors' judgements about included studies. Figure S3: risk of bias summary of authors' judgements about included studies. Figure S4: efficacy of 6 RCTs of MHFZXXD versus western medical therapy. Figure S5: safety of MHFZXXD versus western medical therapy. Figure S6: six months of recurrence of MHFZXXD versus western medical therapy. Table S2: quality of evidence for outcome measure of efficacy. Table S3: quality of evidence for outcome measures of safety. Table S4: quality of evidence for outcome measure of recurrence rate. Table S1: characteristics of included studies. Table S2: characteristics of excluded studies. [file 8132798.f1.zip › 8132798.f1/Table S3 Quality of evidence for outcome measures of safety.docx]

| **Quality assessment** | | | | | | | **Summary of findings** | | | | | **Importance** |
| --- | --- | --- | --- | --- | --- | --- | --- | --- | --- | --- | --- | --- |
|  |  |  |  |  |  |  | **No of patients** | | **Effect** | | **Quality** |  |
| **No of studies** | **Design** | **Limitations** | **Inconsistency** | **Indirectness** | **Imprecision** | **Other considerations** | **Adverse events** | **control** | **Relative (95% CI)** | **Absolute** |  |  |
| **Adverse events** | | | | | | | | | | | | |
| 2 | randomised trials | very serious^1,2^ | no serious inconsistency | no serious indirectness | serious^3^ | reporting bias^4,5^ | 2/103 (1.9%) | 12/103 (11.7%) | RR 0.2 (0.05 to 0.75) | 93 fewer per 1000 (from 29 fewer to 111 fewer) | ⊕OOO VERY LOW | IMPORTANT |
|  |  |  |  |  |  |  |  | 11.8% |  | 94 fewer per 1000 (from 30 fewer to 112 fewer) |  |  |
